# Aiming for a shorter time to diagnosis: pediatric eosinophilic esophagitis in British Columbia

**DOI:** 10.1186/s13223-020-00486-2

**Published:** 2020-10-14

**Authors:** Jocelyn Jia, Edmond S. Chan, Vishal Avinashi, Elaine Hsu, Hin Hin Ko, Lianne Soller

**Affiliations:** 1 Faculty of Medicine, University of Toronto, Toronto, ON Canada; 2 BC Children’s Hospital Research Institute, Vancouver, BC Canada; 3 Division of Allergy and Immunology, Department of Pediatrics, Faculty of Medicine, University of British Columbia, Vancouver, BC Canada; 4 Division of Gastroenterology, Hepatology & Nutrition, Department of Pediatrics, Faculty of Medicine, University of British Columbia, Vancouver, BC Canada; 5 Division of Gastroenterology, Faculty of Medicine, University of British Columbia, Vancouver, BC Canada; 6 St. Paul’s Hospital, Vancouver, BC Canada

**Keywords:** Eosinophilic esophagitis, Pediatric, Diagnosis, Delay

## Abstract

Longer time to diagnosis for patients with eosinophilic esophagitis can lead to adverse patient outcomes, but the length of diagnostic delay has not been quantified for patients with eosinophilic esophagitis in Canada. Our study defines the time to diagnosis (TTD) for pediatric patients with eosinophilic esophagitis in British Columbia and identifies factors that predict increased time to diagnosis. The median TTD was 21 months (1.75 years; IQR = 7, 45) with a median age at EoE diagnosis of 105 months (8.75 years; IQR = 44, 156). Caucasians experienced significantly longer TTD compared to other ethnicities (24 months (IQR = 7, 52) and 12 months (IQR = 4.5, 23) respectively, *p* = 0.008). Caucasian ethnicity (*p* = 0.037) and older age at the time of diagnosis (*p* = 0.006) predicted increased TTD. Our model explained 7.9% (Adjusted R^2^ = 0.079) of the total variance for our cohort.

## Main text

Eosinophilic esophagitis (EoE) is a chronic allergic condition increasing in incidence worldwide, though epidemiological data describing EoE in Canada are lacking [1]. Symptoms of EoE are often nonspecific and can differ by age [1–3], making timely and accurate diagnosis a challenge. No studies have explored time to diagnosis (TTD) for EoE in Canada. Our goals were to describe TTD and factors associated with TTD for children enrolled in the EoE Registry at British Columbia Children’s Hospital (BCCH).

This study was approved by the University of British Columbia Children’s and Women’s Research Ethics Board. BCCH is the sole pediatric tertiary care facility in British Columbia, and is responsible for most pediatric EoE patients in the province. Patients seen in the multidisciplinary EoE clinic—comprised of one pediatric allergist, one pediatric gastroenterologist, and one dietician—at BCCH were eligible for the current study if they were: (1) < 18 years old, (2) resided in BC, (3) had EoE symptoms preceding a biopsy-proven EoE diagnosis (≥ 1 biopsy showing ≥ 15 eosinophils/high-powered field), and (4) consented to participate in the EoE Registry. Data have been entered prospectively into the Registry since 2012 and stored in Research Electronic Data Capture (REDCap), a secure web-based data management software [4].

A cross-sectional analysis using TTD (time between EoE symptom onset and diagnostic endoscopy) as the dependent variable was performed. Independent variables were sex, location (close to tertiary hospital: Vancouver Coastal and Fraser Health Authorities; far from tertiary hospital: Vancouver Island, Interior, and Northern Health Authorities), ethnicity (Caucasian or Other), past history of gastrointestinal (GI) issues (any self-reported GI conditions), past history of allergic conditions (asthma, atopic dermatitis, food allergy, allergic rhinitis, pollen-food syndrome, and/or environmental allergy), family history (first-degree relatives) of GI issues, family history of EoE, and family history of allergic conditions. This information was self-reported by patients or their parent/guardian. Kolmogorov–Smirnov, Mann–Whitney U tests, and Kruskal–Wallis H tests were performed. A multivariable linear regression was performed using TTD as the dependent variable and the following independent variables: sex, location, ethnicity, past history of GI issues, past history of allergic conditions, family history of GI issues, family history of EoE, family history of allergic conditions, and age at diagnosis. SPSS Statistical Package (IBM Corp. Released 2019. IBM SPSS Statistics for Windows, Version 26.0. Armonk, NY: IBM Corp.) was used. Significance was set at *p* < 0.05.

Between July 10th, 2012 and June 8th, 2018, 169 patients were enrolled in the EoE Registry. Patients with missing data (n = 50) or who did not meet the inclusion criteria (n = 4) were excluded, leaving 115 patients for analysis. The study population was 80.0% (92/115) male and 73.9% (85/115) Caucasian with the rest being predominantly South Asian (Table 1). The median age at symptom onset was 72 months (6 years; IQR = 13, 122), the median age at EoE diagnosis was 105 months (8.75 years; IQR = 44, 156), and the median TTD was 21 months (1.75 years; IQR = 7, 45; Table 1).

**Table 1:** Demographic characteristics

Characteristic	Total (n = 115)
Age at symptom onset in months, median (IQR)	72 (13, 122)
Age at diagnosis in months, median (IQR)	105 (44, 156)
TTD in months, median (IQR)	21 (7, 45)
Male	92 (80%)
Close to tertiary hospital (Vancouver Fraser and Vancouver Coastal Health Authorities)	73 (63.5%)
Caucasian^a^	85 (73.9%)
Past history of gastrointestinal issues	44 (38.3%)
Past history of allergic conditions	100 (87%)
Family history of GI issues	58 (50.4%)
Family history of EoE	7 (6.1%)
Family history of allergic conditions	92 (80%)

^a^Non-Caucasians were predominantly South Asian

In our cross-sectional analysis, Caucasians experienced significantly longer TTD compared to other ethnicities (24 months (IQR = 7, 52) and 12 months (IQR = 4.5, 23) respectively, *p* = 0.008, Table 2). No other variables were significant. Multivariate linear regression determined that Caucasian ethnicity (*p* = 0.037) and older age at the time of diagnosis (*p* = 0.006) predicted increased TTD. No other variables were significant. Our model explained 7.9% (Adjusted R^2^ = 0.079) of the total variance for our cohort.

**Table 2:** Cross-sectional analysis

Characteristic	Median time to diagnosis in months (IQR)	*p*-value
Sex		0.469
Male	23 (7, 47.50)	
Female	19 (6, 36)	
Location		0.623
Close to tertiary hospital	21 (9.50, 46.50)	
Far from tertiary hospital	18.50 (4, 37.75)	
Ethnicity		0.008*
Caucasian	24 (7, 52)	
Other	12 (4.50, 23)	
Past history of GI issues		0.798
No	23 (4, 49)	
Yes	18.50 (7, 38.75)	
Past history of allergic conditions		0.917
No	28 (1, 48)	
Yes	19 (7, 44.50)	
Family history of GI issues		0.937
No	21 (8, 43)	
Yes	20 (4, 45.75)	
Family history of EoE		0.977
Yes	15 (4, 52)	
No	21 (7, 44.50)	
Family history of allergic conditions		0.053
No	12 (1, 29)	
Yes	23 (9.25, 47.50)	

*p < 0.05

This is the first study exploring TTD and possible predictors of TTD in a Canadian EoE cohort. Our finding of a TTD of 21 months (IQR = 7, 45) is at the lower end of the range reported in a systematic review for pediatric patients (1.2–3.5 years) in North America and Europe, and much shorter than what has been found for adult EoE patients (3.0–8.0 years). [5] We were surprised that the TTD for our cohort was relatively short, and theorize that referring physicians being aware of our dedicated multidisciplinary clinic for EoE at BCCH may have contributed to decreased TTD through quicker referral times. As far as we know, no other EoE clinics in Canada exist where every patient is interviewed concurrently by a gastroenterologist and an allergist, with both specialists exchanging ideas together with the patient.

Our finding that Caucasians experience longer TTD could be partially explained by racial disparities in EoE presentation between Caucasians and other ethnicities such as South Asians, and suggests that differences in symptom presentation, as well as genetic and/or environmental factors, may influence TTD [6–8]. Given that South Asians are the second largest visible minority group in BC, and the largest in Canada overall, we believe that our results are generalizable to the overall population of Canada, but may not be generalizable to individual provinces or territories with a different distribution of visible minority groups. [9]

Interestingly, older age at diagnosis predicted longer TTD (*p* = 0.006). This is in contrast to a study by Hruz et al. [10]. describing longer TTD in younger EoE patients. Their analysis, however, included mostly adult patients and used discrete age categories instead of a continuous age variable. We hypothesize that parents of older children may perceive their child’s EoE symptoms as less worrisome than parents of younger children and may be less likely to seek medical attention for these symptoms, thereby increasing TTD. Additionally, older children may simply not tell their parents they are experiencing symptoms, or may engage in compensatory behaviours that help minimize their symptoms, which would also increase TTD. Older children may also be more likely to have other medical conditions to which they or their physician may attribute their EoE symptoms, which would increase TTD as well.

A study limitation was the use of patient self-report for certain independent variables. However, misclassification of these self-reported independent variables would have likely been non-differential, and hence should not have affected our results. In addition, our linear regression model explains only 7.9% of the overall variance, indicating that other factors not explored in our cohort have a significant effect on TTD.

In conclusion, our study is the first to explore the possible regional, genetic, and environmental factors that contribute to TTD for EoE patients in a pediatric and Canadian cohort. We found that Caucasians and children diagnosed at older ages experience greater TTD. Although our TTD is at the shorter end of the spectrum, further studies in other Canadian regions, more countries, and the use of objective measures, where possible, are needed to confirm our findings, and to determine strategies to increase EoE awareness and further shorten TTD.

## Data Availability

Summary data and materials are available upon request.

## Abbreviations

EoE: Eosinophilic esophagitis
TTD: Time to diagnosis
BCCH: British Columbia Children’s Hospital
BC: British Columbia
GI: Gastrointestinal
